# Recurrent pericardial effusion after cardiac surgery: the use of colchicine after recalcitrant conventional therapy

**DOI:** 10.1186/1749-8090-6-96

**Published:** 2011-08-10

**Authors:** Luca Dainese, Antioco Cappai, Paolo Biglioli

**Affiliations:** 1 Dpt of Cardiovascular Surgery, University of Milan, Centro Cardiologico Monzino IRCCS, Via Parea 4, 20138 Milan, Italy

## Abstract

Pericardial effusion represents a common postoperative complication in cardiac surgery. Nonetheless, it can be resistant to conventional therapy leading to prolonged in-hospital stay and worsening of clinical conditions.

Recent literature shows that colchicine therapy should be useful in the treatment of recurrent post surgical pericardial effusion.

Hereby we report the case of a patient with postsurgical recurrent effusion treated with colchicine, and a review of literature concerning the use of this old drug.

## Text

Pericardial effusion represents a common postoperative complication in cardiac surgery. Sometimes it can be resistant to conventional therapy leading to prolonged in-hospital stay and worsening of clinical condition.

In the last twenty years colchicine was reported to be clinical effective although most of Authors have published their experience based upon incomplete and sometimes anedoctical experience. Starting from a case of patients successfully treated with this drug we present a review concerning the more recent and statistically based studies about the treatment with this old but actually drug.

### Case Report

A 47-year-old male underwent elective coronary artery bypass grafting. The postoperative intensive care unit stay was uneventful, the postoperative echocardiogram did not showed abnormalities and the patient was discharged on 100 mg cardioaspirin once daily. Five days after the discharge, the patient developed ongoing *dyspnea *and was referred to our department. The echocardiogram demonstrated a large amount of pericardial effusion. Subxiphoid pericardiotomy was performed. The analysis of pericardial fluid excluded the hemorrhagic etiology. Cytology and histology was negative. The drainage catheter was left in place tree days until absence of pericardial effusion was detected by echocardiographyc control.

Nonsteroidal anti-inflammatory drugs (NSAIDs) and corticosteroids were initiated. After two weeks the echocardiographic control revealed a new onset pericardial effusion that was again treated surgically.

Considering the failure of the conventional therapy, the patient was treated with 2 mg/die colchicine for 1 month followed by 1 mg/die for a further 6 months, without recurrence of the effusion after follow-up of 6 months. No side-effects were observed.

## Discussion

Postpericardiotomy syndrome (PPS) is a frequent complication of cardiac operations affecting from 20 to 40% of patients appearing within 6 months of the initial operation with a median of 4 weeks after heart surgery [1-5]. The PPS is acutely provoked by a greater antiheart antibody response (antisarcolemmal and antifibrillary) and appear to be variants of a common immunopathic process [1-5].

Clinically is characterized by fever, *eosinophilia*, and pleuritis. Additional findings include *malaise*, splinter hemorrhages, leukocytosis, and an increased erythrocyte sedimentation rate. Some patients have mild normocytic anemia. Liver function test results are normal and chest radiography typically shows a characteristic bilateral pleural effusion.

Standard pericardial effusion treatment usually consists of administration of aspirin or nonsteroidal anti-inflammatory drugs (NSAIDs) to decrease the fever and the chest pain. Although most patients respond to nonsteroidal anti-inflammatory drugs or corticosteroids [4-8] the failure of this treatment is possible leading to complications including cardiac tamponade, constrictive pericarditis, and occlusion of the coronary artery bypass graft. In the last few years, colchicine has appeared as an useful medical treatment for recurrent pericardial effusion. Although the mechanism is not fully understood [6-8] this drug seems to be safe and effective.

Nowadays there is not uniformity on the dosage, the duration of the therapy after the release of symptoms, expecially after surgical procedure (Table 1).

**Table 1:** dose of colchicine in pericardial effusion

Autors	Loading Dose	Maintenance	Journal and year	NSAIDs or Corticosteroids
Horneffer PJ et al.	2 mg/day for 1 or 2 days	1 mg/day for several weeks or months	*J Thor Cardiov Surgery 1990 and ESC 2004*	

Imazio et al.	1 or 2 mg/day (0.5 or 1 mg/day < 70 kg)	0.5 or 1.0 mg/day for 3 months	*Circulation 2005*	Aspirin 800 mg every 6 or 8 hours for 7 or 10 days (tapering for 3-4 weeks)

Spodick DH (Permayner-Miranda)	0.6 mg twice daily	0.6 mg twice daily for 1 or 2 weeks and tapering for 1 or 2 weeks	*JAMA 2003*	Ibuprofen 800 mg every 8 hours

Millaire A et Al	3 mg/day	1 mg/day (1 to 27 months -mean 7.7)	*Eur Heart J 1994*	

Guindo J et Al	1 mg/day	1 mg/day	*Circulation 1990*	Prednisone 20-60 mg/day

Lange RA	0.6 mg twice daily	0.6 mg twice daily	*NEJM 2004*	Aspirin 2-4 mg/daily or Indometacin 75-225 mg/daily or Ibuprofene 1600-3200 mg/daily

Adler Y et Al	1 mg/day	1 mg/day	*Am J Cardiol 1994*	

De la Serna R et al	1 mg/day	1 mg/day	*Lancet 1987*	

Adler Y et Al.		1 mg/day (1 or 6 months)	*Clin Cardiol 1998*	Aspirin

Adler Y et Al.	2-3 mg/day (unclear)	1 mg/day at least 1 year	*Circulation 1998*	Ibuprofen

Imazio M et Al. CORE	1.0-2.0 mg/day	0.5-1.0 mg/day for 6 months	*Arch Intern Med 2005*	Aspirin 800 mg every 6 or 8 hours for 7 or 10 days (tapering for 3-4 weeks)

Maisch B et Al.	2 mg/day	1 mg/day	*Eur Heart J 2004*	

Shabetai R	2 mg/day	1 mg/day		

Soler-Soler J et Al	2 mg/day	1 mg/day	*Heart 2004*	

Imazio M et Al. COPPS (Prevention PPS)	1.0-2.0 mg/day first day (1 mg/d < 70 kg)	0.5-1.0 mg/day for 1 month (0.5 mg/d < 70 kg)	*Int J Cardiology 2006*	

Little and Freeman	2 mg/day	1 mg/day for 3 months	Circulation 2006	Aspirin 650-975 mg every 6-8 h for 4 weeks

Imazio et Al.	1 mg/day	1 mg/day	Eur Heart Journal 2003	Aspirin 600-800 mg every 6 or 8 hours for 7 or 10 days (tapering for 2-3 weeks)

Sagristà-Sauleda et Al	1-2 mg	0.5-1 mg/day for 1 year	Rev Esp Cardiol 2005	

Finkelstein et Al. (Prevention PPS)		1.5 mg every 8 h/day for 1 month	Herz 2002	

Imazio et Al.	2 mg	1 mg/day for 6-12 months	J Cardiov Medicine 2007	

Artom et Al.	1 mg	1 mg	Eu Heart Journal 2005	

Colchicine is a tricyclic alkaloid, and its pain-relieving and anti-inflammatory effects were linked to its ability to bind with tubulin inhibiting neutrophil motility and activity, leading to a net anti-inflammatory effect. The main anti-inflammatory mechanism of colchicine is via inhibition of granulocyte migration into the inflamed area inhibiting mitosis and affecting cells with high turnover (GI tract, marrow) [1-5]. Thus it inhibits various leukocyte functions and depresses the action of the leukocytes and of the fibroblasts at the site of the inflammation [1-5]. The most common side effects of colchicine involve the stomach and bowel and are dose related including nausea, vomiting, abdominal pain, and *diarrhea*.

Only in the past five years some Authors [4,9-14] published the first multicenter result of use of colchicine in pericardiac effusion based upon not only *anedoctal *experiences. Specifically postsurgical studies use are laking.

As a matter of fact the use of colchicine for pericardial effusion start in 1987 when three patients were treated for recurrences of acute pericarditis in with colchicine (1 mg/day) with no relapses during a follow up period of 15-35 months [6]. Even if some Authors [10] recommend the use of the drug at the first recurrence as adjunct to conventional therapy, others propose to consider the drug only after failure of conventional therapies for the second or subsequent recurrence [4,12,13].

Adler and collegues [8] suggested that the dose of colchicine is 1 mg/d for at least 1 year, with a gradual tapering off. The need for a loading dose of 2 to 3 mg/d at the beginning of treatment is unclear. Guindo et al. [11] observed that colchicine (1 mg/day) is effective in relieving pain and preventing recurrent pericarditis treating a few number of patient treated also with prednisone (10-60 mg/day).

On the contrary Millaire and colleagues [9] used colchicine in recurrent pericarditis in nineteen consecutive patients with recurrent pericarditis (two episodes or more). Colchicine was given at a loading dose of 3 mg and a maintenance dose of 1 mg daily for 1-27 months with benefit and resolution of pericarditis in 14 patients. The authors concluded that colchicine offered a very good benefit/risk ratio without the need to use corticosteroids treatment. Guindo et Al. [7] in its more extensive experience (51 patients) treated with corticosteroids and NSAIDs or *pericardiocentesis *conclude that recurrences were generally minor and controlled with restitution if the patient were treated with colchicine therapy (loading dose 0.5-3 mg/day with maintenance dose 0.5-2 mg/day). This results are confirmed by the Colchicine for Acute Pericarditis (COPE) Trial [11] concerning the use of colchicine in different type of pericardic syndrome also post pericardiectomy. One hundred twenty patients with a first episode of acute pericarditis (idiopathic, acute, postpericardiotomy syndrome and connective tissue disease) entered a randomized, open-label trial comparing aspirin plus colchicine (1.0 to 2.0 mg for the first day followed by 0.5 to 1.0 mg/d for 3 months) with treatment with aspirin alone. Even if colchicine reduced symptoms at 72 hours and recurrence at 18 months it was discontinued in 5 patients because of *diarrhea*. No other adverse events were noted. None of 120 patients developed cardiac tamponade or progressed to pericardial constriction.

In the 2004 guidelines of the European Society of Cardiology [4] suggested colchicine as a possible therapeutic choice in acute pericarditis or for the treatment and prevention of recurrent pericarditis (RP) after failure of conventional therapies. The dose recommanded for RP is 2 mg/day for one or two day followed by 1 mg/day. The dose for initial attack or prevention of recurrences is 0.5 mg bid. In PPS the colchicine is recommanded for several weeks or months, even after disapparence of effusion. Imazio and collegues [11,13,15] in several papers specify that the dose for prevention of PPS is 1.0 to 2.0 mg for the first day followed by a maintenance dose of 0.5 to 1.0 mg daily for 1 month for patients ≥ 70 kg. A lower dose (initial dose: 1.0 mg and maintenance dose 0.5 mg daily) is given to patients < 70 kg or intolerant to the highest dose (initial dose 1.0 mg BID and maintenance dose of 0.5 mg BID).

The same dose is given in the CORE study [10], but the time of administration is 6 months. Sagrista et Al. [12] recommended starting dose of 1 mg every 12 hours (the dose may be reduced to 0.5 every 12 hours in patient with digestive intolerance). The duration of treatment with colchicine is 1 year (0.5 mg- 1 mg/day).

In 2007 Imazio et Al. [13] recommend dose is 2 mg/day for one-two days, followed by a maintenance dose of 1 mg/day (0.5 mg twice daily). At doses of 1-2 mg/day, colchicine is well tolerated even when given continuously over decades. Even if Spodick et Al. [16] advises the best therapy for the acute and also for recurrent pericarditis combined ibuprofen (800 mg every 8 hours) with colchicine (0.6 mg twice daily) for 7 to 14 days followed by tapering for another 1 or 2 weeks Artom et al. [14] found that treatment with colchicine is highly effective in preventing recurrent pericarditis, while pretreatment with corticosteroids exacerbates and extends the course of recurrent pericarditis. Comparing two surgical group pretreated with colchicine (1.5 mg/day for one month) and placebo Finkelstein Y et al. [17] found significantly less occurrence of pericardial effusion in first group (10% vs. 20%).

Considering the different experiences above mentioned we give to the patient a 2 mg/die dose of colchicine for 1 month followed by 1 mg/die for a further 6 months without echocardiographyc signs of recurrent pericardial effusion.

Recent literature shows that colchicine therapy should be usefull in treatment of recurrent post surgical pericardial effusion.

Neverthless post surgical large studies are necessary to state definitely the use of colchicine therapy in recurrent postsurgical pericardial effusion.

## Competing interests

The authors declare that they have no competing interests.

## Authors' contributions

LD, AC, PB wrote the article.

All authors read and approved the article.
